# On Dit

**Published:** 1876-01

**Authors:** 


### ON DIT.

Dr. Holden, of Newark, N. J., says, in the *New York Medical:* It is now eight years since a physician, who had been fifty years in practice, suggested for a patient of mine, suffering from ulcerative stomatitis, a mixture of Peruvian bark, acacia, and sugar, made into a paste with cream; its success then led me to use it in one case after another, with the same result; and in scarlatina, diphtheria, and all aphthous affections, it has proved invaluable. The following is the formula: ℞.—Cort. Peruv. flav., two drams; Acaciæ pulv., one dram; Sacch. alb., half dram. M. Sig. Mix one half of this powder in a tablespoonful of cream, and apply frequently with a camel's hair brush.

In *post-nasal catarrh,* Dr. Beverly Robinson recommends: Powdered cubebs, two ounces; sirup of orange, three ounces; mint water, eight ounces. Of this half a teaspoonful is to be administered every three or four hours. It is necessary to continue the cubebs for one or two months, and by its use the sensation of stuffiness in the head and hawking mucus is relieved.

Dr. John Hughes Bennett, of Edinburgh, holds: 1st. Chloral mitigates the effect of strychnia, so that life may be saved by chloral after a fatal dose of strychnia, but strychnia is less likely to save life after a fatal dose of chloral. 2d. The antagonism between sulphate of atropia and Calabar-bean is slight. 3d. The effects of Calabar-bean are considerably modified by chloral, if the latter substance is given before the former. Hydro-chlorate and meconate of morphia in no way antagonize Calabar-bean. 5. Sulphate of atropia is antagonistic to meconate of morphia within a limited area, but the limit is too narrow for practical service. 6. Tea, coffee, theine, caffeine, and guaranine, are somewhat antagonistic to meconate of morphia. 7. Strychnia and

Calabar-bean modified the action of each other, but neither saved life after a fatal dose of the other. 8. Bromal hydrate and atropine are antagonistic, but, while atropia may save life after a fatal dose of the bromal, the converse, apparently, does not hold good.

Mr. Peyrand, of Bordeaux, recommends the powdered bromide of potassium as a caustic, and at the same time anæsthetic in ulcerated cancer.

Dr. H. Nickell, in the *Buffalo Medical*, highly commends the following formula in chorea: ℞.—Strychniæ Sulph., half grain; Acid. acet., dilut. sufficient quantity; Ferri pyrophos., one and a half dram; Cinnamon water, three ounces; Extr. belladonna, two grains; Syr. cort. aurant., one ounce; Liquor potass. arsen., one and a half fluid dram. M. Sig. Shake well, and give a teaspoonful three times daily, after meals.

J. H. Salls gives, in *American Journal Pharmacy*, as the result of chemical analysis of Winslow's Soothing Syrup, a morphia strength of a little over a quarter grain to the ounce of syrup.

In the same journal, Charles F. Hartwig commends the use of an ordinary glass female syringe to expedite the formation of emulsions. The principle is that of the sillabub churn, and the syringe is operated in a similar manner.

Dr. Hughlings Jackson, of the London Hospital, says, in the *Lancet*, "I do not see how the diagnosis that there is actual softening of the brain is in any case to be possibly arrived at, *unless the patient has certain local paralytic symptoms, as hemiplegia, or some other symptoms implying a local cerebral lesion, such as affection of speech;* or, again, unless there be signs of cerebral tumor (severe headache, urgent vomiting, and double optic neuritis), or evidence of injury to the head. For, so far as I know, cerebral softening is always local; I know nothing of general or universal softening of the brain."

In the *Medical News*, Dr. Thackeray, of Dakota, commends the use of cider vinegar as a local remedy in the pruritis formicans of pregnancy.

Dr. Slane, of Goalpera, thinks well of the carbazotate of ammonia as a substitute for quinia in intermittents. He states that the largest quantity given was eight grains in the day. It does not increase the appetite; it produces tinnitus aurium and slight deafness; the urine is tinged of a deep orange color, which soon passes off; the skin and conjunctiva acquire a jaundiced hue whilst under its influence only; it is extremely bitter to the taste, and is eligibly exhibited in pill.

In the *Boston Medical,* Dr. Hubbell, of Troy, N. Y., relates a case of empyema, in a child of three and a half years, treated by free incission (one inch and a half) and washing out daily the chest. The result was prompt and striking, and the recovery complete, with perfect restoration of function to the lung.

Mr. Callender reports to the Clinical Society of London his experience with salicylic acid as a surgical dressing of wounds. He finds a solution of more than two parts in the hundred act as an irritant to wounds, and causes constitutional disturbance. He rates its antiseptic power below that of carbolic acid, and the healing is less kindly and less rapid.

Who Owns the Prescription?—The *Pharmacal Gazette* answers: "There is little doubt that this advice, like any other property or commodity, would be found to belong to the man who paid for it. * * * If physicians would not have their patients know what medicine they are taking, they should furnish the medicine instead of the formula; and if they would prevent valuable prescriptions from being used by the friends of their patients, the only sure way is not to write out the formulas and sell them." That editor's head is level; and moreover he's honest, as an editor ought to be. Let medical men take due notice, and govern themselves accordingly.

Dr. Grailey Hewitt considers, in the *Lancet,* the subject of convulsions, as connected with disorders of the unimpregnated uterus. He concludes that these reflex convulsions attend especially upon flexions of the uterus, and are to be cured by appropriate treatment of the flexions.

Mr. Henry Smith, of King's College Hospital, gives a second series of cases of hemorrhoids treated by his clamp and cautery. In his four hundred cases he has had four deaths. There has been no case of pyæmia; but two of erysipelas, one of them narrowly escaping fatal issue. Of hemorrhage he remarks that in no instance has it been so formidable as to necessitate plugging of the rectum; in eight of his cases the bleeding has, however, required "very active measures" for its control. This he attributes to want of proper care in the use of the cautery. In three cases he has had stricture to result; this he attributes to too free removal of skin. In one case anal fistula resulted from the operation.

In the Huntingdon County Hospital, Mr. Holderness, in amputating an arm with Esmarch's bandage, met with accidental hemorrhage of severe character, in consequence of the biceps muscle, when cut, retracting from beneath the elastic and liberating the brachial artery from pressure. Care should be used to place the elastic sufficient distance above the line of incission to avoid such accident.

